# Preclinical evaluation and reverse phase protein Array-based profiling of PI3K and MEK inhibitors in endometrial carcinoma in vitro

**DOI:** 10.1186/s12885-018-4035-0

**Published:** 2018-02-09

**Authors:** Ozlem Aslan, Mattia Cremona, Clare Morgan, Lydia W. Cheung, Gordon B. Mills, Bryan T. Hennessy

**Affiliations:** 1 Department of Medical Oncology, Royal College of Surgeons in Ireland, Beaumont Hospital, Dublin 9, Ireland; 2 Department of Systems Biology, the University of Texas M.D. Anderson Cancer Center, Houston, TX 77030 USA

**Keywords:** Endometrial cancer, Biomarkers, PTEN loss, *PIK3CA*, *KRAS*, PI3K inhibitor, MEK inhibitor, Protein signalling

## Abstract

**Background:**

The phosphoinositide-3-kinase (PI3K) pathway is the most commonly activated pathway in cancers due to mutations at multiple nodes and loss of PTEN. Furthermore, in endometrial cancer (EC), PI3K and RAS/RAF/MEK/MAPK (RAS/MAPK herein) pathway mutations frequently co-exist. We examined the role of PI3K and RAS/MAPK pathway mutations in determining responsiveness to therapies targeted to these pathways in vitro in EC.

**Methods:**

13 EC cell lines were profiled for their PI3K pathway and *KRAS* mutational and PTEN protein status and treated with one MEK- and two PI3K- targeted inhibitors alone and in combination. Expression and phosphorylation of 66 proteins were evaluated by Reverse-Phase-Protein-Array (RPPA) in 6 EC cell lines to identify signalling changes in these pathways in response to therapy.

**Results:**

PTEN protein loss and the absence of any tested pathway mutations are dominant negative predictors of sensitivity to MEK inhibition. *KRAS*-mutated cells were most sensitive to MEK inhibition, but significantly more resistant to PI3K inhibition than *KRAS*-wild-type cell lines. Combinations of PI3K and MEK inhibitors showed synergy or additivity in all but two cell lines tested. Treatment of *KRAS*-mutated cells with PI3K inhibitors and treatment of PTEN-low cells with a MEK inhibitor were most likely to induce activation of MEK/MAPK and AKT, respectively, likely indicative of feedback-loop regulation.

**Conclusions:**

MEK inhibition may be a promising treatment modality, not just for ECs with mutated *KRAS*, but also for those with retained PTEN. Up-regulation of MEK/MAPK signalling by PI3K inhibition, and up-regulation of AKT activation by MEK inhibition may serve as potential biomarkers of likely responsiveness to each inhibitor.

**Electronic supplementary material:**

The online version of this article (10.1186/s12885-018-4035-0) contains supplementary material, which is available to authorized users.

## Background

The phosphoinositide 3-kinase (PI3K) pathway is commonly activated in many cancer types, including EC, frequently due to mutations at multiple nodes, for example *PIK3CA* (which encodes the p110α catalytic subunit of PI3K), or loss of PTEN protein expression (the major tumour suppressor that regulates this pathway). The RAS/MAPK pathway has also been shown to be a good target for novel therapy in preclinical cancer models. Additionally, bidirectional crosstalk with the PI3K pathway [1, 2] suggests that interactions between these two pathways might dictate the responsiveness of cancer cells to inhibitors targeted against these pathways [3]. We have previously performed an integrated analysis of the PI3K and interacting pathways in 243 endometrial cancer and showed that the PI3K pathway is activated in over 80% of endometrioid endometrial cancers by mutations in the *PTEN* (44%), *PIK3CA* (40%), *PIK3R1* (20%) and *PIK3R2* (5%) genes [4, 5]. In terms of the RAS/MAPK pathway, we found activating *KRAS* mutations in approximately 20% of endometrial cancers [6, 7]. The coexistence of mutations in *KRAS* and PI3K pathway members suggests that the RAS/MAPK and PI3K pathways may interact as critical drivers of pathogenesis in EC and serve as targets for the development of novel cancer treatments. However, the implications of these mutations for EC responsiveness to therapies targeted to these pathways have not been fully defined.

Novel inhibitors targeting the PI3K pathway and RAS/MAPK pathway (e.g. MEK inhibitors) are currently being developed. Pictilisib (GDC-0941) and apitolisib (GDC-0980) are potent inhibitors of class I PI3K isoforms [8], class I PI3K and mTOR-kinase (TORC1/2), respectively [9, 10], and cobimetinib (GDC-0973) is a selective small-molecule inhibitor of MEK that is highly potent [11]. Targeting both pathways has been shown to be more effective in preclinical cancer models than targeting either pathway alone [11–13]. Here, we investigated the effects of mutations in the PI3K and RAS/MAPK pathways on tumour cell signalling and on responsiveness to these PI3K and RAS/MAPK-targeted therapies alone and in combination in EC.

## Methods

### EC cell lines and cell culture

The human endometrial cancer cell lines AN3CA, HEC1A, HEC1B, HEC50, ECC1, EFE184, ETN1, KLE and SKUT2 were obtained from the Department of Systems Biology, the University of Texas M.D. Anderson Cancer Center (Houston, USA), EN, MFE296 and MFE280 cell lines were purchased from the German Collection of Microorganisms and Cell Cultures (DSMZ; Braunschweig, Germany). EN-1078D cell line was kindly provided by Prof Eric Asselin (University of Quebec), where informed consent was attained and research studies approved by the Montreal University Institutional Review Board [14]. The EC cell lines AN3CA, HEC1A, KLE and SKUT2 cell lines were grown in DMEM-F12 (Sigma #D6421), with 10% FBS (Lonza #DE14-801F); EN-1078D was grown in DMEM-F12 (Sigma #D6421) and 10% FBS with the addition of 50 μg/mL gentamicin (Invitrogen #15710–049), HEC50 was grown in DMEM-F12 with the addition of 2 mM glutamine (Sigma #D8437) and 10% FBS. ECC1, EFE184, EN, ETN1 and MFE280 were grown in RPMI-1640 (Sigma #R8758) supplemented with 10% FBS. MFE296 was grown in RPMI-1640 (Sigma #R8758) with 10% FBS and insulin-transferrin-selenium-X (Invitrogen #51500). HEC1B and HEC59 were grown in MEM (Sigma # 2279) with the addition of 10% FBS, 1% non-essential amino acids and 10 mL glutamine (Sigma #D8437). Growth media were supplemented with 100 μg/ml streptomycin and 100 U/ml penicillin (Invitrogen) and all cell lines were cultured at 37 °C in 5% CO_2_. Cell lines were fingerprinted at the beginning of the study to confirm their identities.

### Cell line somatic mutation genotyping

Genomic DNA was extracted using QIAamp DNA Mini Kit (Qiagen). We designed high-throughput assays for somatic mutations in *KRAS, PIK3CA, PIK3R1, PIK3R2* and *PTEN,* and applied a mass spectroscopy–based approach to detect single nucleotide polymorphisms (MassARRAY, Sequenom) as described previously [5, 15].

### Cell viability assays

The inhibitors pictilisib (GDC-0941), apitolisib (GDC-0980) and cobimetinib (GDC-0973) were obtained from Genentech, Inc. 1 × 10^4^ cells/well were plated into flat-bottomed, 96-well plates and allowed to attach overnight. All drug treatments including combinations were tested in triplicate during a 5-day incubation period with serial dilutions of drug in a final volume of 200 uL. Drug-free controls were included in each assay. DMSO controls were also performed for each assay. Plates were incubated at 37 °C in a humidified atmosphere with 5% CO_2_ and cell viability was determined using an acid phosphatase assay as described previously [16, 17].

### Protein extraction and reverse phase protein Array (RPPA)

RPPA is a high through-put antibody based technique used for functional proteomic assessments of a large number of tumour samples and it offers a platform for comparison of the relative protein expression between these samples [18]. We plated cells in 6-well plates, allowed them to reach 60–80% confluence and extracted their proteins as described by us and others previously [15, 19, 20]. Cells were washed with cold PBS and lysed with ice-cold lysis buffer (1% Triton X-100, 50 mm HEPES, pH 7.4, 150 mM NaCl, 1.5 mM MgCl2, 1 mM EGTA, 100 mM NaF, 10 mM Na pyrophosphate, 1 mM Na3VO4, 10% glycerol) supplemented with proteinase inhibitors (Roche Applied Science, Indianapolis, IN) as described previously [19, 21].

For RPPA, four-fold serial dilutions of protein extracts from EC cell lines were performed. Serially diluted lysates were arrayed on Oncyte Avid nitrocellulose-coated slides (Grace Bio-Labs, Bend, OR) using a QArray 2 arrayer (Molecular Devices, Wokingham, UK). Diluted samples were robotically printed on multiple slides that included positive and negative controls. Slides were probed with primary antibodies (see Additional file 1: Table S1) followed by a secondary antibody – either goat anti-rabbit IgG (1:5000) (Vector Laboratories, Burlingame, CA) or rabbit anti-mouse IgG (1:10) (Dako) depending on the particular primary antibody. Signals were amplified using a Dakocytomation-catalysed system (Dako) and visualized by chromogenic detection (using diaminobenzidine). Slides were then scanned, analysed, and quantified using customized software (Microvigene, VigeneTech Inc.) to generate spot intensities.

Heat-maps were generated from normalized data using unsupervised hierarchical clustering analysis performed with publicly available Morpheus software (https://software.broadinstitute.org/morpheus/).

### Statistical analysis

Drug combination assays synergy was assessed using the combination index method [22] using CompuSyn software (Combosyn Inc., Paramus, NJ, USA) [12]. A combination index (CI) of < 1 is considered to indicate synergy, = 1 is considered additive and > 1 is considered antagonistic [22]. We applied the Student’s t-test to test the significance of pair-wise comparisons using GraphPad-Prism.5. *P-*value < 0.05 was considered statistically significant.

## Results

### Genomic and proteomic features of the endometrial cancer cell lines

By applying a mass spectroscopy–based analysis (MassARRAY, Sequenom) to the 13 EC cell lines we confirmed their mutational status. As we and others have previously shown in primary endometrioid endometrial carcinomas (EECs) [23, 24], we found that multiple members of the PI3K pathway and sometimes *KRAS* were mutated in the same cell lines, including concomitant mutations (Tables 1 and 2).

**Table 1:** IC_50_s of endometrial cancer cell lines (*n* = 13) to the PI3Ki pictilisib, PI3K/mTORi apitolisib and the MEKi cobimetinib as single agents (n = 13), and the combination of pictilisib with cobimetinib and apitolisib with cobimetinib (*n* = 11)

Cell Line	Mutational status	PTEN expression	PI3Ki Pictilisib (uM)	PI3K/mTORi Apitolisib (uM)	MEKi Cobimetinib (uM)	Combination of Pictilisib:Cobimetinib (uM)		Combination of Apitolisib:Cobimetinib (uM)	
			IC_50_	IC_50_	IC_50_	IC_50_	CI at ED_75_	IC50	CI at ED_75_
SKUT2	*PIK3CA* only	High	0.413	0.196	0.101	0.096	0.138	0.049	0.152
MFE280	*PIK3CA* only	High	0.440	0.247	0.461	0.207	0.357	0.091	0.528
EN1078D	*PTEN + PIK3R1*	High	0.242	0.079	0.178	0.103	0.634	0.057	0.468
MFE296	*PTEN + PIK3CA*	High	0.273	0.106	0.301	0.383	1.037	0.120	0.650
ETN1	*PTEN*	High	0.544	0.387	1.077	0.174	0.426	0.177	0.495
AN3CA	*PTEN + PIK3R1*	Low	0.057	0.028	0.518	0.339	0.129	0.070	0.142
HEC59	*PTEN + PIK3R1*	Low	0.189	0.018	3.289	–	–	–	–
EN	*PTEN + PIK3CA + PIK3R1*	Low	1.597	0.548	**36.511**	3.117	**1.555**	2.175	**1.200**
HEC1A	*KRAS + PIK3CA*	High	2.054	0.214	0.012	–	–	–	–
HEC1B	*KRAS + PIK3CA*	High	0.798	0.704	0.033	0.050	0.031	0.054	0.035
HEC50	*KRAS + PIK3R1*	High	0.247	0.210	**0.001**	0.131	**4.921**	0.169	**6.613**
EFE184	WT for all	High	0.372	0.368	0.149	0.074	0.433	0.157	0.328
KLE	WT for all	High	2.796	7.253	39.337	0.644	0.142	0.144	0.005

Drug combinations exhibiting antagonistic effects (CI values > 1) are in shown bold

*IC*_50_ inhibitor concentration to inhibit 50% of the cell growth, *PI3Ki* PI3K inhibitor, *PI3K/mTORi* PI3K/mTOR inhibitor, *MEKi* MEK inhibitor, *CI* Combination Index, *ED*_75_ Effective Dose exhibiting 75% of loss in cell viability

**Table 2:** Classification of endometrial cancer cell lines into four groups

Groups	Cell line	*PIK3CA*	*KRAS*	*PTEN*	*PIK3R1*	*PIK3R2*	PTEN expression
Group 1 (PIK3CA-mutated only)	SKUT2	E545K (hetero)	Wild-type	Wild-type	Wild-type	Wild-type	High
	MFE280	H1047Y (hetero)	Wild-type	Wild-type	Wild-type	Wild-type	High
Group 2a (*PTEN*-mutated, PTEN-retained)	**EN1078D**	Wild-type	Wild-type	Y88C	G376R (hetero)	Wild-type	High
	MFE296	P539R (hetero); I20M (hetero)	Wild-type	R130Q (homo); N322 fs (hetero)	Wild-type	Wild-type	High
	**ETN1**	Wild-type	Wild-type	I122V (hetero), R130L (hetero); N328 fs (hetero)	Wild-type	Wild-type	High
Group 2b (*PTEN*-mutated and PTEN-Loss)	AN3CA	Wild-type	Wild-type	R130fs (homo)	REID557del (hetero)	Wild-type	Low
	HEC59	Wild-type	Wild-type	Y46H (hetero); R233 (hetero); P246L (hetero)	T473S (hetero); K567E (hetero); S460 fs (hetero)	Wild-type	Low
	EN	T1025A (hetero)	Wild-type	K266 fs (hetero)	N260S (hetero)	Wild-type	Low
Group 3 (*KRAS*-mutated)	HEC1A	G1049R (hetero)	G12D (homo)	Wild-type	Wild-type	Wild-type	High
	**HEC1B**	G1049R (hetero)	G12D (homo)	Wild-type	Wild-type	Wild-type	High
	**HEC50**	Wild-type	G12D (hetero)	Wild-type	E468InsGEYDRLYE (homo)	Wild-type	High
Group 4 (Wild-type)	EFE184	Wild-type	Wild-type	Wild-type	Wild-type	Wild-type	High
	**KLE**	Wild-type	Wild-type	Wild-type	Wild-type	Wild-type	High

The 6 cell lines highlighted in bold were selected for further RPPA analyses

Further, using RPPA, we found that PTEN protein expression was retained in the majority of the EC cell lines with the exception of three cell lines (EN, AN3CA, HEC59) which are all *PTEN*-mutated and had relatively high levels of phosphorylated AKT. We observed that the presence of a *PTEN* mutation was significantly associated with activation of PI3K pathway as determined by high levels of AKT phosphorylation at Thr^308^ (*P* = 0.005) and at Ser^473^ (*P* = 0.008) in EC cell lines, however no significant association was observed between *PIK3CA, PIK3R1* or *KRAS* mutation and AKT phosphorylation levels (see Additional file 2: Table S2). These results are inconsistent with previous studies in breast cancer cell lines [15] and endometrial cancer cell lines [24], providing *PTEN* mutation or PTEN loss as important activators of PI3K-mediated pro-survival signalling through AKT.

**Fig. 1:**
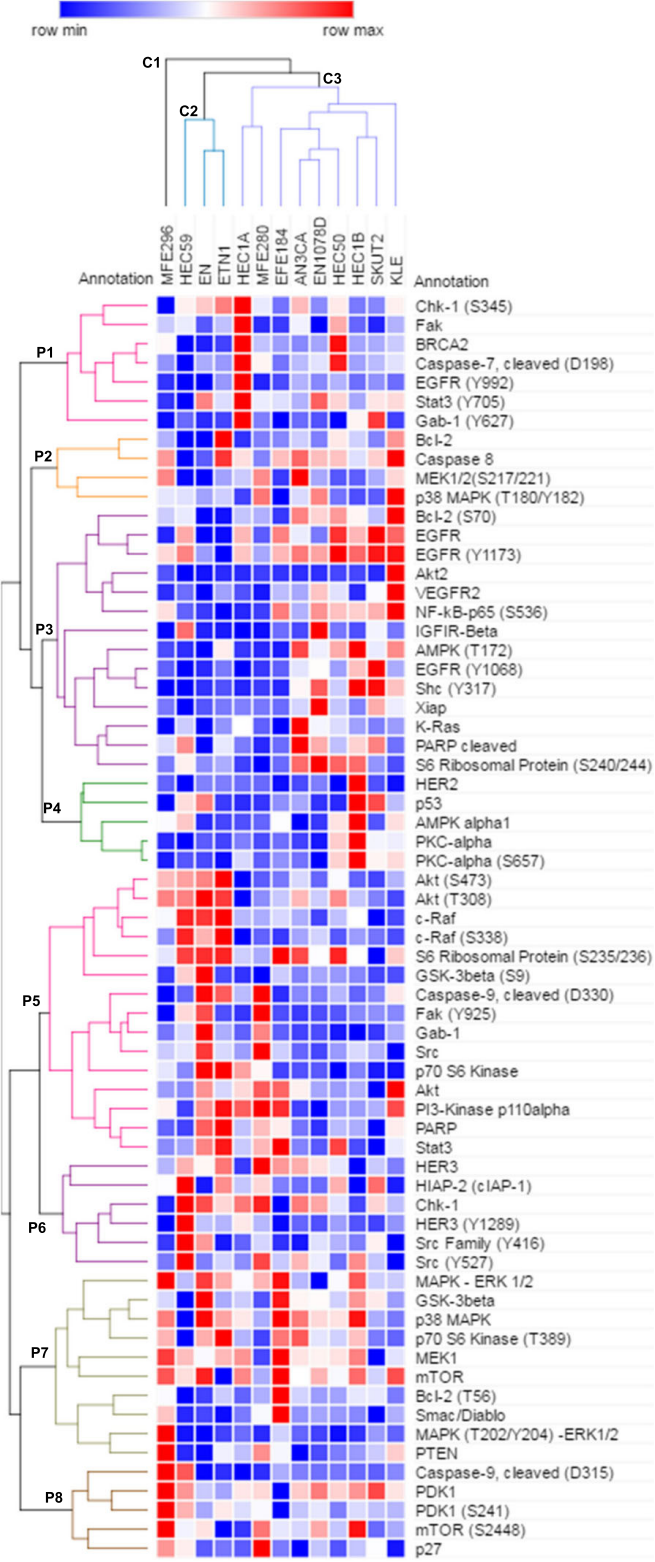
Heat-maps showing baseline RPPA data of 13 EC cell lines using 66 proteins. Signal intensities were normalized and used for an unsupervised cluster analysis. Three cell lines clusters C1, C2 and C3 and eight protein clusters P1 to P8 were identified

Unsupervised hierarchical clustering revealed three major cell line clusters (designated as C1, C2 and C3) (Fig. 1). Cluster C1 contained one cell line only, the *PTEN*-mutated MFE296, distinguished by high levels of PTEN, total MAPK-ERK1/2 expression and MAPK-ERK1/2 phosphorylation at Thr^202^/Tyr^204^. Cluster C2 and C3 were composed of four (HEC1A, MFE280, EFE184, KLE) and five (AN3CA, EN-1078D, HEC1B, HEC50, SKUT2) cell lines, respectively (Fig. 1). Cell line cluster C2 was composed of three of the *PTEN*-mutated cell lines (EN, HEC59, ETN1) of which two expressed low levels of PTEN (EN, HEC59). As compared with cluster C3, cell lines in cluster C2 contained higher levels of higher levels of phosphorylated AKT at Ser^473^ and Thr^308^, phosphorylated c-Raf at Ser^338^, phosphorylated S6 ribosomal protein at Ser^235/236^ and P70 S6 kinase, and lower levels of phosphorylated NFkβ p65^Ser536^, phosphorylated MEK1/2^Ser217/221^, phosphorylated MAPK-ERK1/2^Thr202/Tyr204^ and phosphorylated GSK-3β^Ser9^. Thus it seems that cluster C2 is defined by activation of the PI3K pathway and cluster C3 by activation of the RAS/MAPK pathway. Unsupervised hierarchical clustering of the 66 proteins also revealed eight protein clusters (designated P1 to P8) (Fig. 1).

### Sensitivity of endometrial cancer cell lines to two PI3K inhibitors and a MEK inhibitor

We observed that EC cell lines possessing a *KRAS* mutation (HEC50, HEC1A and HEC1B) were more sensitive to the MEK inhibitor cobimetinib (IC_50_s from 0.001 to 0.033 μM) when compared with the other cell lines tested (IC_50_s from 0.101 to 39.337 μM) (Table 1). Conversely, EC cell lines possessing a *PTEN* mutation (MFE296, EN-1078D, ETN1, AN3CA, HEC59, EN) were more resistant to MEK inhibition (IC_50_s from 0.178 to 36.511 μM) when compared with the cell lines without a *PTEN* mutation (IC_50_s from 0.101 to 0.001 μM (with the exception of the wild-type cell line KLE with a IC_50_ of 39.337 μM).

In terms of the dual inhibition of PI3K and mTOR, we observed that EC cell lines with coexisting *PTEN* and *PIK3R1* mutations (HEC59, AN3CA and EN-1078D) were more sensitive to the PI3K/mTOR inhibitor apitolisib (IC_50_s from 0.018 to 0.079 μM) when compared with the others (IC_50_s from 0.196 to 7.253 μM) (Table 1). Two wild-type cell lines (EFE184, KLE) showed different responses to all three inhibitors, whilst KLE was very resistant to pictilisib, apitolisib and cobimetinib treatments (IC_50_s 2.796, 7.253 and 39.337 μM, respectively), the other wild-type cell line EFE184 was markedly sensitive with the IC_50_s of 0.372, 0.368 and 0.149 μM for pictilisib, apitolisib and cobimetinib, respectively (Table 1).

**Fig. 2:**
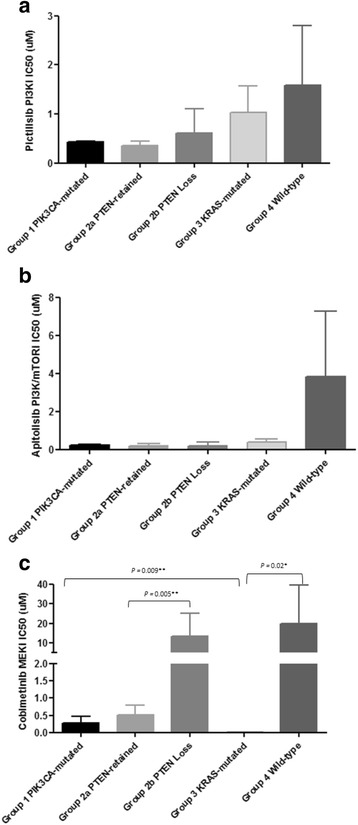
Comparative inhibitor responsiveness between endometrial cancer cell line groups are shown in Table 2. The figures derived by comparing average IC_50_s between the groups for each inhibitor alone and in combination **a** Comparative responsiveness of pictilisib in EC cell line groups, **b** Comparative responsiveness of apitolisib in EC cell line groups, **c** Comparative responsiveness of cobimetinib in EC cell line groups. *P* < 0.05 is statistically significant

Next, we examined if expression levels of PTEN protein play an important role in response to therapies targeted to the PI3K and MEK pathways. Thus, we compared EC cell lines with PTEN protein loss (AN3CA, EN and HEC59) against EC cell lines with retained PTEN protein, and found that cell lines exhibiting PTEN loss were more sensitive to the PI3K inhibitor pictilisib (IC_50_s from 0.057 to 1.597 μM) when compared with cell lines with retained PTEN expression (IC_50_s from 0.242 to 2.796 μM). In terms of apitolisib sensitivity, cell lines with PTEN loss were more sensitive (IC_50_s from 0.018 to 0.548 μM) when compared with PTEN retained cell lines (IC_50_s from 0.106 to 7.253 μM). However, these cell lines with PTEN loss were more resistant to MEK inhibition (IC_50_s from 0.518 to 36.511 μM) when compared with cell lines with retained PTEN expression (IC_50_s from 0.001 to 1.077 μM), (again with the exception of the MEK inhibitor resistant wild-type KLE cell line with high levels of PTEN), indicating PTEN loss as a dominant marker of sensitivity to PI3K inhibition and resistance to MEK inhibition (Fig. 2c).

### Sensitivity of EC cell lines to the two PI3K inhibitors and a MEK inhibitor in combination

Next, we sought to examine whether dual inhibition of both the PI3K and MEK pathways might result in synergistic effects on cell viability. We found that the combination of pictilisib with cobimetinib, and the combination of apitolisib with cobimetinib both resulted in synergistic growth inhibition in most of the cell lines tested (2/10), with the exception of 2 cell lines (EN, HEC50) (Table 1, Fig. 4a–b). Endometrial cancer cell lines in which synergism was observed to the combination of pictilisib (PI3Ki), apitolisib (PI3K/mTORi) and cobimetinib (MEKi) are shown in Additional file 3: Figure S1.

We observed strong antagonism as indicated by CI values greater than 1 (see Materials and Methods), for EN cell line with the combination of pictilisib and cobimetinib (CI value = 1.555), and with the combination of apitolisib and cobimetinib (CI value = 1.200). This antagonism may occur due to the high resistance to the MEK inhibitor cobimetinib in this cell line (IC_50_: 36.511 μM) when compared with pictilisib and apitolisib therapies alone (IC_50_s 1.597 and 0.548 μM, respectively) (Table 1). We also determined strong antagonism for HEC50 cell line with the combination of pictilisib and cobimetinib, and with the combination of apitolisib and cobimetinib (CI values 4.921 and 6.613, respectively). In contrast to EN cell line, antagonism in HEC50 cell line may occur due to the high sensitivity to MEK inhibition (IC_50:_ 0.001 μM) (Table 1).

**Fig. 3:**
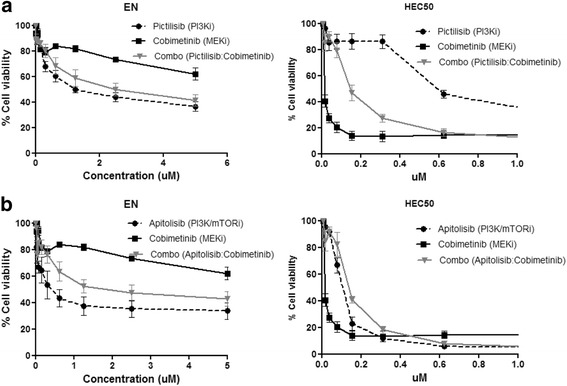
Endometrial cancer cell lines in which antagonism was observed to the combination of pictilisib (PI3Ki), apitolisib (PI3K/mTORi) and cobimetinib (MEKi). **a** Two cell lines in which antagonism was observed to the combination of PI3K and MEK inhibition. **b** Two cell lines in which antagonism was observed to the combination of PI3K/mTOR and MEK inhibition

HEC50 and EN cell lines in which antagonism was observed to the combination of pictilisib (PI3Ki), apitolisib (PI3K/mTORi) and cobimetinib (MEKi) are shown in Fig. 3.

### Classification of endometrial cancer cell lines

We detected differential responsiveness to the PI3K inhibitors and to the MEK inhibitor between the cell lines with the presence of *PTEN* mutations and PTEN loss being significantly associated with PI3K pathway activation, enhanced sensitivity to PI3K inhibition and remarkable resistance to the MEK inhibitor. Additionally, based on proteomics profiling, cell lines in cluster C2 (HEC59, EN, ETN1) were more resistant to the MEK inhibitor cobimetinib (IC_50_s 3.289, 36.511 and 1.077 μM, respectively) than cell lines in cluster C3 (IC_50_s from 0.001 to 0.518; *P* = 0.04), yet again suggesting that *PTEN* mutations and the level of PTEN protein expression are important events in cell responsiveness to therapies targeted to the PI3K and RAS/MAPK pathways. Therefore, based on differential sensitivity to the PI3K and MEK inhibitors and differential activation of the PI3K and RAS/MAPK signalling pathways associated with the clustering in Fig. 1b which seems to be largely driven by *PTEN*, *PIK3CA* and *KRAS* mutations and PTEN protein levels, we classified our cell lines into these 4 variables as follows:

- Group 1 (*PIK3CA* mutated EC cell lines): SKUT2, MFE280.
- Group 2 (*PTEN* mutated EC cell lines):
  - (*PTEN* mutated EC cells with retained PTEN): EN-1078D, MFE296, ETN1.
  - (*PTEN* mutated EC cells with PTEN Loss): AN3CA, HEC59, EN.
- Group 3 (*KRAS* mutated EC cells): HEC1A, HEC1B, HEC50.
- Group 4 (wild-type EC cells): EFE184, KLE (Table 2 and Fig. 2).

In this classification system, *PTEN* mutations regarded as dominant over *PIK3CA* mutations because they, and not *PIK3CA* mutations, are associated with AKT activation as shown earlier. The comparative responsiveness of each group to the three targeted inhibitors is shown in Fig. 2. Comparison of these groups revealed that group 2a and group 2b cell lines with *PTEN* mutations were most sensitive to the PI3K inhibitor pictilisib, followed by group 1 cell lines with *PIK3CA* mutations only (IC_50_ = 0.43 ± 0.01). In the *PTEN*-mutated group 2 cell lines, pictilisib responsiveness was independent of PTEN protein expression status. *KRAS*-mutated cell lines in group 3 and wild-type cell lines in group 4were the most resistant to this inhibitor (IC_50_ values 1.03 ± 0.53 and 1.58 ± 1.21, respectively). Group 1 and group 4 cell lines did not differ significantly in their sensitivities to pictilisib, despite the former having and the latter lacking *PIK3CA* mutations.

Responses to the PI3K/mTOR inhibitor apitolisib overall resembled those to the PI3K inhibitor pictilisib, with for example *PTEN*-mutated cell lines with retained PTEN (group 2a) were being more sensitive (IC50 = 0.17 ± 0.05) compared to wild-type cell lines in group 4 (IC_50_ = 0.33 ± 0.06) (Fig. 2b).However, group 3 *KRAS*-mutated cell lines were more sensitive to this inhibitor (IC_50_ = 0.19 ± 0.09 μM) compared with group 4 cell lines (IC_50_ = 3.81 ± 3.44 μM). In general, the differences between the groups in terms of apitolisib sensitivity were not as marked as the differences between the groups in terms of pictilisib sensitivity.

In terms of MEK inhibition, cobimetinib induced a strong inhibition in all except group 2b and group 4 EC cell lines (IC_50_ = 10.74 ± 4.47 μM and 1.28 ± 0.60 μM, respectively), particularly in group 1 cell lines with *PIK3CA* mutations (IC_50_ = 0.31 ± 0.08 μM) and group 3 *KRAS*-mutated cell lines (IC_50_ = 0.05 ± 0.01 μM) (Fig. 2c). However, responses to MEK inhibition varied noticeably between *PTEN*-mutated cell lines with retained PTEN protein in group 2a (IC_50_ = 0.31 ± 0.08 μM) and *PTEN*-mutated cell lines exhibiting loss of PTEN protein in group 2b (IC_50_ = 10.74 ± 4.47 μM).

### Proteomic effects in endometrial cancer cell lines of PI3K inhibitors and a MEK inhibitor

Next, we sought to determine how PI3K and MEK inhibitors affect protein signalling in EC cell lines. We selected a group of 6 EC cell lines, one cell line from the *PIK3CA*-mutated group 1 (SKUT2), one cell line from the *PTEN*-mutated PTEN retained cell lines in group 2a (EN-1078D), one cell line from the group 2b cell lines with PTEN loss (AN3CA), two cell lines from the *KRAS*-mutated group 3 (HEC1B; –*PIK3CA*-mutated, and HEC50; –*PIK3CA*-wild-type), and finally one cell line from the wild-type group (KLE).

**Fig. 4:**
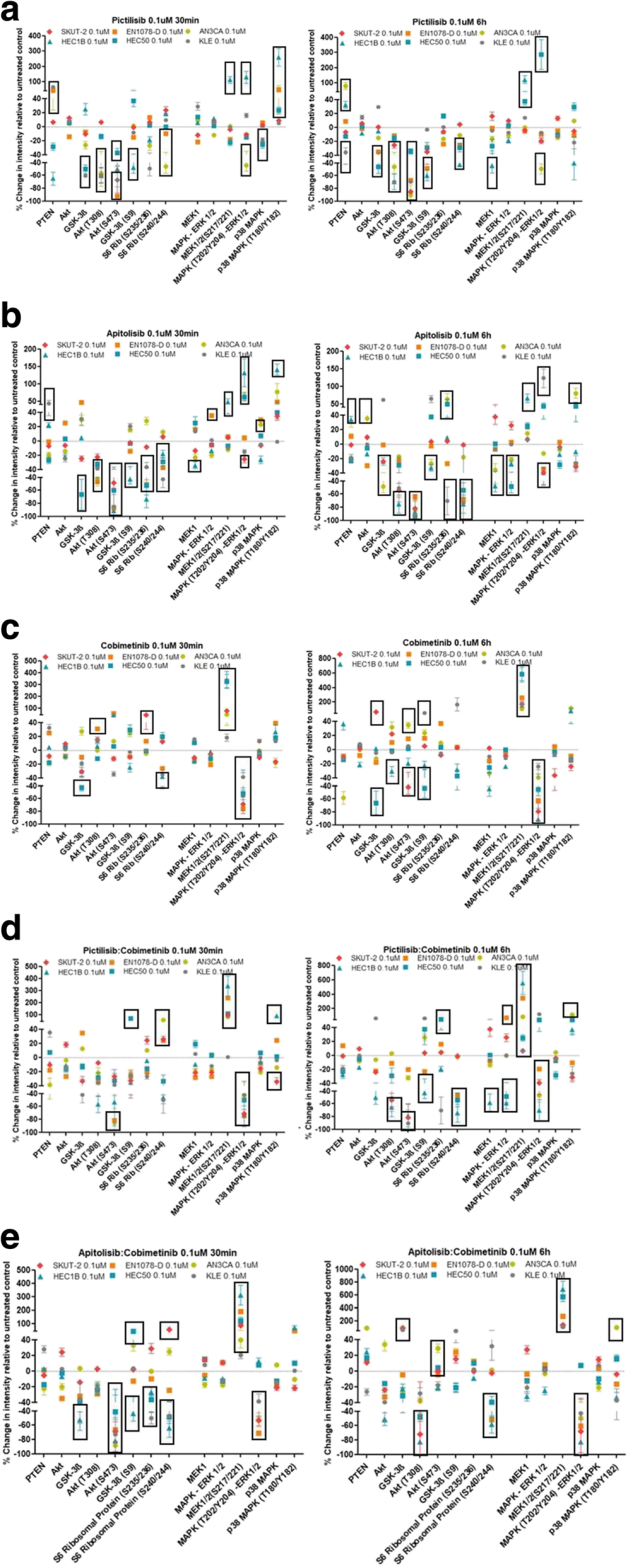
Control-normalized changes in the expression and phosphorylation of PI3K and MAPK signalling proteins induced by 0.1 μM concentration at two different time points of the PI3K inhibitors **a** pictilisib at 30 min and 6 h, **b** apitolisib at 30 min and 6 h, **c** cobimetinib at 30 min and 6 h, **d** the combination of pictilisib with cobimetinib at 30 min and 6 h, and **e** the combination of apitolisib with cobimetinib at 30 min and 6 h. The specific phosphorylation sites evaluated are indicated (e.g. AKT (T308) in AKT phosphorylation at threonine 308). Cell lines displaying significant differential changes (*P* < 0.05) in protein expression and phosphorylation are shown in boxes

The data in Fig. 4a–e demonstrate changes in phosphorylation and expression of AKT and RAS/MAPK signalling proteins after treatment with 0.1 μM of the PI3K, PI3K/mTOR and MEK inhibitors, and the combinations for 30 min and 6 h in 6 EC cell lines. Similar to observation in patients [25], we observed decreases in phosphorylation of AKT following treatment with the PI3K inhibitors pictilisib and apitolisib when compared with control-treated cells. We also observed that reductions in AKT, S6 ribosomal protein and GSK-3β phosphorylation occurred in some cell lines. Figure 4c also demonstrates a general decrease in the phosphorylation of MAPK-ERK1/2^Thr202/Tyr204^ after treatment with the MEK inhibitor.

We found exposure time to be an important variable, particularly in some EC cells, with some pathways up-regulated immediately after drug exposure down-regulated at longer exposure. For example, 30 min exposure to each of the PI3K inhibitors and the MEK inhibitor resulted in up-regulation of p38 MAPK^Thr180/Tyr182^ phosphorylation in the *PTEN* mutated, PTEN retained cell line EN-1078D, with down-regulation of p38 MAPK^Thr180/Tyr182^ phosphorylation at longer exposure (Fig. 4a–c).

While treatment with the PI3K inhibitor pictilisib significantly suppressed the levels of phosphorylated S6 ribosomal protein (especially at Ser^240/244^), phosphorylated AKT (at Thr^308^ and Ser^473^) and phosphorylated GSK-3-β^Ser9^ in most EC cell lines compared with control-treated cells (Fig. 4a), it significantly induced phosphorylation of MEK1/2^Ser217/221^, MAPK^Thr202/Tyr204^ and p38^Thr180/Tyr182^ in some cell lines, most notably in the *KRAS*-mutated cell lines HEC1B and HEC50 at most time point, a likely sign of a feedback loop regulation. While this occurred after 30 min in HEC1B, it was more obvious after 6 h in HEC50, possibly indicating different adaptations by feedback regulation (Fig. 4a). This data suggests that pictilisib-induced activation of the RAS/MAPK pathway is most likely to occur in *KRAS*-mutated cell lines, possibly underlying the resistance of these cell lines to this PI3K inhibitor.

Consistent with growth inhibition responses to the PI3K/mTOR inhibitor apitolisib resembling those to the PI3K inhibitor pictilisib in the cell line panel, the protein signalling effects of these inhibitors were also very comparable. In general, this inhibitor led to inhibition of AKT phosphorylation in the cell lines in comparison with control-treated cells. As with pictilisib, there was no obvious correlation between degree of the PI3K pathway inhibition and apitolisib sensitivity. Apitolisib induced increases in MEK, MAPK and p38 MAPK phosphorylation were also observed, in particular at 30 min in the *KRAS*-mutated cell lines. Of the 6 cell lines tested herein, AN3CA (PTEN-low) and KLE (wild-type) were the most resistant to the MEK inhibitor cobimetinib. While this inhibitor decreased MAPK phosphorylation at Thr^202^/Tyr^204^ in all cell lines (Fig. 4c), its effect seemed to be least marked in these two cell lines. Further, at 6 h, cobimetinib treatment was associated with increased phosphorylation of AKT^Ser473^ (in AN3CA) and of GSK-β^Ser9^ and S6 ribosomal protein^Ser240/244^ (in KLE). While MEK phosphorylation at Ser^217/221^ was in general increased in response to cobimetinib, this effect was most marked in the two most sensitive and *KRAS*-mutated cell lines HEC1B and HEC50 (Fig. 4c). This increase in MEK phosphorylation is likely to be a feedback loop-induced mechanism that seems most marked in the presence of mutated *KRAS*.

Dual blockage of the PI3K and RAS/MAPK pathways has been shown to synergistically inhibit tumour cell growth in different cancers [11, 26, 27]; including endometrial cancer [28, 29]. Indeed, we observed that treatment with PI3K and MEK inhibitor combinations was more effective than each inhibitor alone in most EC cell lines tested (9 out of 11) (Table 1). While the PI3K and MEK inhibitor combinations did not abrogate MEK activation induced by cobimetinib alone, they did abrogate the increases in AKT (e.g. in AN3CA) and S6 ribosomal protein^Ser240/244^ (e.g. in KLE) phosphorylation seen with the MEK inhibitor alone (Fig. 4d), possibly contributing to the synergy observed in these cell lines. In HEC50, we also observed that the significant inhibition of MAPK^Thr202/Tyr204^ phosphorylation induced by cobimetinib was no longer present with the pictilisib:cobimetinib and apitolisib:cobimetinib combinations, especially at 6 h (Fig. 4d), possibly contributing to the antagonism seen in this cell line with the drug combinations.

## Discussion

In this study, we profiled the responses of a diverse set of endometrial cancer cell lines to PI3K and MEK inhibitors alone and in combination, to characterise variability in responses by genotype. We found that, in agreement with previous studies [5, 24], mutations in multiple members of the PI3K pathway and *KRAS* were co-existed in several endometrial cancer cell lines, including concomitant mutations in the PI3K pathway. Several studies have shown that the correlation between mutations in some PI3K pathway members and sensitivity to PI3K inhibitors is weak [30–32].

Classification of tumours based on their genetic signatures and responses to inhibitors is a major goal for advancing targeted personalized therapies. Recent studies proposed to classify endometrial cancers into 4 categories: POLE ultra-mutated, microsatellite instability hyper-mutated, copy number-low, and copy number-high [9, 33]. In this study, we focused on the PI3K and RAS/MAPK pathway genomic and proteomic status of EC cell lines and assessed their sensitivity to three selective inhibitors of the PI3K and RAS/MAPK pathways alone and in combination.

We detected differential responsiveness to the PI3K inhibitors and to the MEK inhibitor between the cell lines, with the presence of *PTEN* mutations and PTEN loss being significantly associated with PI3K pathway activation and enhanced sensitivity to PI3K inhibition and remarkable resistance to the MEK inhibitor. We also examined if proteomic clustering can be used to identify subgroups of cell lines with differential responses to the different targeted therapies.

Indeed, we observed no significant association between *PIK3CA* or *PIK3R1* mutations and AKT phosphorylation, in contrast to PTEN loss and *PTEN* mutations which were significantly associated with PI3K pathway activation as determined by high levels of AKT phosphorylation. These results are consistent with the previous study by Weigelt et al. [24] and suggest that *PTEN* mutations and PTEN loss rather than *PIK3CA* mutations are associated with increased AKT activation in endometrial cancer. Possibly in line with this, we found in our study that *PTEN* mutations and PTEN loss are dominant predictors of sensitivity to the PI3K inhibitor pictilisib, while *PIK3R1*, *PIK3CA* and *KRAS* are not. Indeed, in our panel of EC cell lines, *PIK3CA* and *KRAS* mutations were associated with relative resistance to PI3K inhibitors. While the three *KRAS*-mutated cell lines were most sensitive to the MEK inhibitor cobimetinib, *PTEN* mutations and, in particular PTEN loss, were also dominant biomarkers of resistance to this inhibitor. Our data here suggests a key role for PTEN protein loss in mediating resistance to MEK inhibition, perhaps mediated by a feed-back-loop as suggested previously [13]. This is consistent with the previous study by Hoeflich et al. [13], that also showed loss of PTEN protein to be a negative predictor of response to MEK inhibition.

RPPA analysis revealed that PI3K inhibitors (pictilisib and apitolisib) inhibit AKT phosphorylation in most of the cell lines after 30 min treatment as expected. However, in *KRAS*-mutated cell lines, treatment with PI3K inhibitors results in an increase in MEK and MAPK phosphorylation, indicative of a potential link between PI3K inhibition and feedback activation of the RAS/MAPK pathway in these cells, which may contribute to cell line resistance to pictilisib. Thus, PI3K inhibitor-induced RAS/MAPK pathway activation in some cells may explain the ineffectiveness of single agentPI3K inhibition in inhibiting cell growth concurrent targeting of both pathways.

Dual inhibition of the PI3K and RAS/MAPK pathways has been shown to provide a more effective treatment strategy than inhibition of one pathway. In this study, the combinations of the PI3K inhibitor with the MEK inhibitor and of the PI3K/mTOR inhibitor with the MEK inhibitor, showed additivity or synergy in inhibiting the growth of most of the cell lines tested, with the exception of two cell lines. One of these cell lines had a *KRAS* mutation only without a PI3K pathway mutation (HEC50), and the antagonism of the combinations may possibly be partly as a result of the particularly marked anti-tumour efficacy of MEK inhibition alone in this cell line. Our RPPA data showed that treatment of HEC50 cells with the MEK inhibitor alone inhibited MAPK phosphorylation more effectively than the combination of the PI3K and MEK inhibitors, possibly contributing to this antagonism. Indeed, with the combination treatments, MAPK phosphorylation at Thr^202^/Tyr^204^ was not significantly inhibited in HEC50, in contrast to the effect of cobimetinib alone. This data suggests that dual blockade of PI3K with MEK may not improve the efficacy of MEK inhibition in some *KRAS*-mutated EC cell lines that do not possess a PI3K pathway mutation.

In terms of developing a classification system that integrates PI3K and RAS/MAPK genomic events to predict the likelihood of inhibitor response, the dominance of *PTEN* mutations and PTEN protein loss as predictors of AKT activation and drug response, make it reasonable to center classification surrounding these events. As *KRAS* mutations are also associated with resistance to PI3K inhibition and other targeted therapies (e.g. EGFR), we felt it is reasonable to define another cell line subgroup based on these mutations. This left four cell lines, two with *PIK3CA* mutations only, and two with no mutations in *PIK3CA*, *KRAS*, *PTEN*, *PIK3R1*or *PIK3R2*, which were subdivided into *PIK3CA* mutation alone and wild-type subgroups. Overall, this classification system, shown in Table 2, offers the potential to classify endometrial cancers based on responsiveness to PI3K and MEK inhibitors.

## Conclusion

In summary, inhibition of MEK with cobimetinib alone or in combination with PI3K inhibitors (pictilisib and apitolisib) could be a promising treatment modality, not just for endometrial cancer patients with mutated *KRAS*, but also for those with retained PTEN expression. Up-regulation of MEK/MAPK signalling by PI3K inhibition, and up-regulation of AKT activation by MEK inhibition may serve as potential biomarkers of likely responsiveness to each inhibitor. Combinations of PI3K and MEK inhibitors are potentially promising for the treatment of many ECs.

## Additional files

Additional file 1: Table S1. The 66 primary antibodies used in RPPA in this study are listed. The phosphorylation sites (amino acids) for phospho-specific antibodies are shown. (XLSX 10 kb)

Additional file 2: Table S2. Baseline levels of 13 EC cell lines for the 66 primary antibodies used in RPPA are listed and t-test result for the comparison between PTEN-mutated EC and wild-type-PTEN EC cell lines shown. Significant associations are in highlighted in pink. (XLSX 21 kb)

Additional file 3: Figure S1. Endometrial cancer cell lines in which synergism was observed to the combination of pictilisib (PI3K), apitolisib (PI3K/mTOR), and cobimetinib (MEK) inhibitors. a) Eight cell lines in which synergism was observed to the combination of PI3K and MEK inhibition. b) Eight cell lines in which synergism was observed to the combination of PI3K/mTOR and MEK inhibition. (XLSX 158 kb)

## Abbreviations

AKT: Also known as protein kinase B, serine/threonine-specific protein kinase
CI: Combination index
DMEM: Dulbecco’s modified eagle medium
DMSO: Dimethyl sulfoxide
DNA: Deoxyribonucleic acid
EC: Endometrial cancer
ED75: Effective dose exhibiting 75% of loss in cell viability
FBS: Fetal bovine serum
HEPES: 4-(2-hydroxyethyl)-1-piperazineethanesulfonic acid
IC50: Half maximal inhibitory concentration
*KRAS*: Kirsten ras oncogene homolog
MEK: Also known as MAPK, Mitogen-activated protein kinase
MEKi: MEK inhibitor
MEM: Minimum essential medium
mTOR: Mechanistic target of rapamycin
PBS: Phosphate buffered saline
PI3K: Phosphoinositide-3-kinase
PI3K/mTORi: PI3K/mTOR inhibitor
PI3Ki: PI3K inhibitor
*PIK3CA*: Phosphatidylinositol-4,5-bisphosphate 3-kinase catalytic subunit alpha
*PIK3R1*: Phosphoinositide-3-kinase regulatory subunit 1
*PIK3R2*: Phosphoinositide-3-kinase regulatory subunit 2
PTEN: Phosphatase and tensin homolog
RAS/MAPK: Mitogen-activated protein kinase pathway
RPMI: Roswell Park Memorial Institute medium
RPPA: Reverse phase protein array
SEM: Standard error of mean
